# Maternal Gestational Diabetes Mellitus increases placental and foetal lipoprotein-associated Phospholipase A2 which might exert protective functions against oxidative stress

**DOI:** 10.1038/s41598-017-13051-6

**Published:** 2017-10-03

**Authors:** Carolin Schliefsteiner, Birgit Hirschmugl, Susanne Kopp, Sanja Curcic, Eva Maria Bernhart, Gunther Marsche, Uwe Lang, Gernot Desoye, Christian Wadsack

**Affiliations:** 1 Department of Obstetrics and Gynaecology, Medical University of Graz, Graz, Austria; 2 Department of Clinical and Experimental Pharmacology, Medical University of Graz, Graz, Austria; 3 Institute of Molecular Biology and Biochemistry, Medical University of Graz, Graz, Austria; 4 BioTechMed-Graz, Graz, Austria

## Abstract

Increased Lipoprotein associated phospholipase A_2_ (LpPLA_2_) has been associated with inflammatory pathologies, including Type 2 Diabetes. Studies on LpPLA_2_ and Gestational Diabetes Mellitus (GDM) are rare, and have focused mostly on maternal outcome. In the present study, we investigated whether LpPLA_2_ activity on foetal lipoproteins is altered by maternal GDM and/or obesity (a major risk factor for GDM), thereby contributing to changes in lipoprotein functionality. We identified HDL as the major carrier of LpPLA_2_ activity in the foetus, which is in contrast to adults. We observed marked expression of LpPLA_2_ in placental macrophages (Hofbauer cells; HBCs) and found that LpPLA_2_ activity in these cells was increased by insulin, leptin, and pro-inflammatory cytokines. These regulators were also increased in plasma of children born from GDM pregnancies. Our results suggest that insulin, leptin, and pro-inflammatory cytokines are positive regulators of LpPLA_2_ activity in the foeto-placental unit. Of particular interest, functional assays using a specific LpPLA_2_ inhibitor suggest that high-density lipoprotein (HDL)-associated LpPLA_2_ exerts anti-oxidative, athero-protective functions on placental endothelium and foetus. Our results therefore raise the possibility that foetal HDL-associated LpPLA_2_ might act as an anti-inflammatory enzyme improving vascular barrier function.

## Introduction

Although pathologies such as atherosclerosis and diabetes are generally associated with derangements in lipid and glucose metabolism, these conditions are also characterized by inflammation and vascular dysfunction (for extensive review see^1,2^).

One potent inflammatory mediator is the platelet-activating factor (PAF, 1-O-alkyl-2-acetyl-sn-glycero-3-phosphocholine) which is produced by a wide range of cells, including endothelial cells, macrophages and neutrophils. The biological functions of PAF and structurally related oxidized phospholipids (oxPL) as bioactive ligands are mediated through a G-protein coupled receptor, the PAF receptor (PAFR). This leads to the production of cytokines, leukotrienes, and prostaglandins either locally or even systemically, thereby driving the pro-inflammatory immune response in conditions such as e.g. asthma, septic shock, and allergic responses^3^. Lipoprotein-associated phospholipase A_2_ (LpPLA_2_) is a member of the PLA_2_ superfamily with a unique substrate preference for PAF and oxPL. The enzyme is synthesized almost exclusively by macrophages^4,5^ and circulates within the blood bound to low density lipoprotein (LDL, 80%) and high density lipoprotein (HDL, 20%) in adults.

LpPLA_2_ activity and mass are both elevated in numerous pathologies, all of which are associated with inflammation, e.g. hypercholesterolemia^6^, atherosclerosis^7^, diabetes^8^, and metabolic syndrome^9^. However, LpPLA_2_ deficiency, common in about 5% of the Japanese population, has been shown to further exacerbate conditions like asthma^10^ and atherosclerosis^11^. Therefore, low - but not absent - levels of LpPLA_2_ have been considered beneficial, whereas higher levels are detrimental to maintaining homeostasis against inflammation. Recently, controversy about LpPLA_2_ activities has been raised as LpPLA_2_ action releases lysophosphatidylcholine (lysoPC) and oxidized non-esterified fatty acids (oxNEFAs) from oxPL, both metabolites showing pro- as well as anti-inflammatory activities^12,13^. Current discussion centres around the specific role of LpPLA_2_ as a driver, an inhibitor, or a mere biomarker of inflammation (for review see^14,15^). Despite large clinical studies showing correlation of LpPLA_2_ mass and/or activity with parameters such as body-mass index (BMI), insulin resistance and pancreatic-beta cell function^16–19^, knowledge about factors regulating LpPLA_2_ activity using *in vitro* cell culture models is limited.

Gestational diabetes mellitus (GDM) is a form of diabetes first recognized in the second trimester of pregnancy. Maternal insulin resistance is a physiological process developing during gestation to ensure the foetal energy supply. Whereas most women can cope with this metabolic adaptation, some develop GDM. In Europe, prevalence for GDM is around five percent^20^. However, prevalence is steadily increasing, as maternal obesity is a major predisposing factor for GDM and more women of child-bearing age are overweight or obese^21^.

Apart from hyperglycemia, GDM is a condition associated with increased oxidative stress and inflammation in placenta and foetus^22,23^. GDM also increases trans-placental fatty acid and lipoprotein transport and turnover^24,25^. Previous research of our group showed that GDM causes major changes in the composition of the foetal lipoprotein proteome^26^ and that foetal lipoprotein functionality is different from adults^27^.

Knowledge about the role of LpPLA_2_ in human pregnancy is limited. LpPLA_2_ is also referred to as plasma platelet activating factor acetyl hydrolase, or plasma PAF-AH. Furthermore, two intracellular cytosolic PAF-AH forms exist, PAF-AH I and PAF-AH II. The latter shares significant sequence homology with LpPLA_2_^28^. Animal studies, as well as a limited number of human studies, have indicated that the balance between PAF and intracellular PAF-AH in uterine and foetal cells, but also maternal plasma LpPLA_2_, is important during the implantation phase of the embryo, as well as during parturition^29,30^. LpPLA_2_ levels in pregnant women are lower than in non-pregnant women, and steadily decline through the course of gestation in rabbits^31^, and humans^32^. This is regulated by the rising oestrogen level during pregnancy^33^. In pregnancies complicated by hypertension, LpPLA_2_ levels remain unchanged^34^ or are even elevated^35^ compared to non-pregnant controls.

In pregnancies affected by GDM, maternal serum LpPLA_2_ levels are elevated compared to healthy pregnant women^36^ at time of delivery; no data on LpPLA_2_ levels throughout gestation in GDM pregnancies exist. In GDM mothers, LpPLA_2_ activity was associated with triglyceride levels, ApoB levels and LDL-C levels in multivariate regression analysis^36^.

Persistently elevated maternal LpPLA_2_ serum levels can be found even two years after a GDM pregnancy, and correlate with the risk for metabolic syndrome^37^. This is remarkable as GDM is a transient form of hyperglycaemia that usually disappears after delivery.

**Figure 1:**
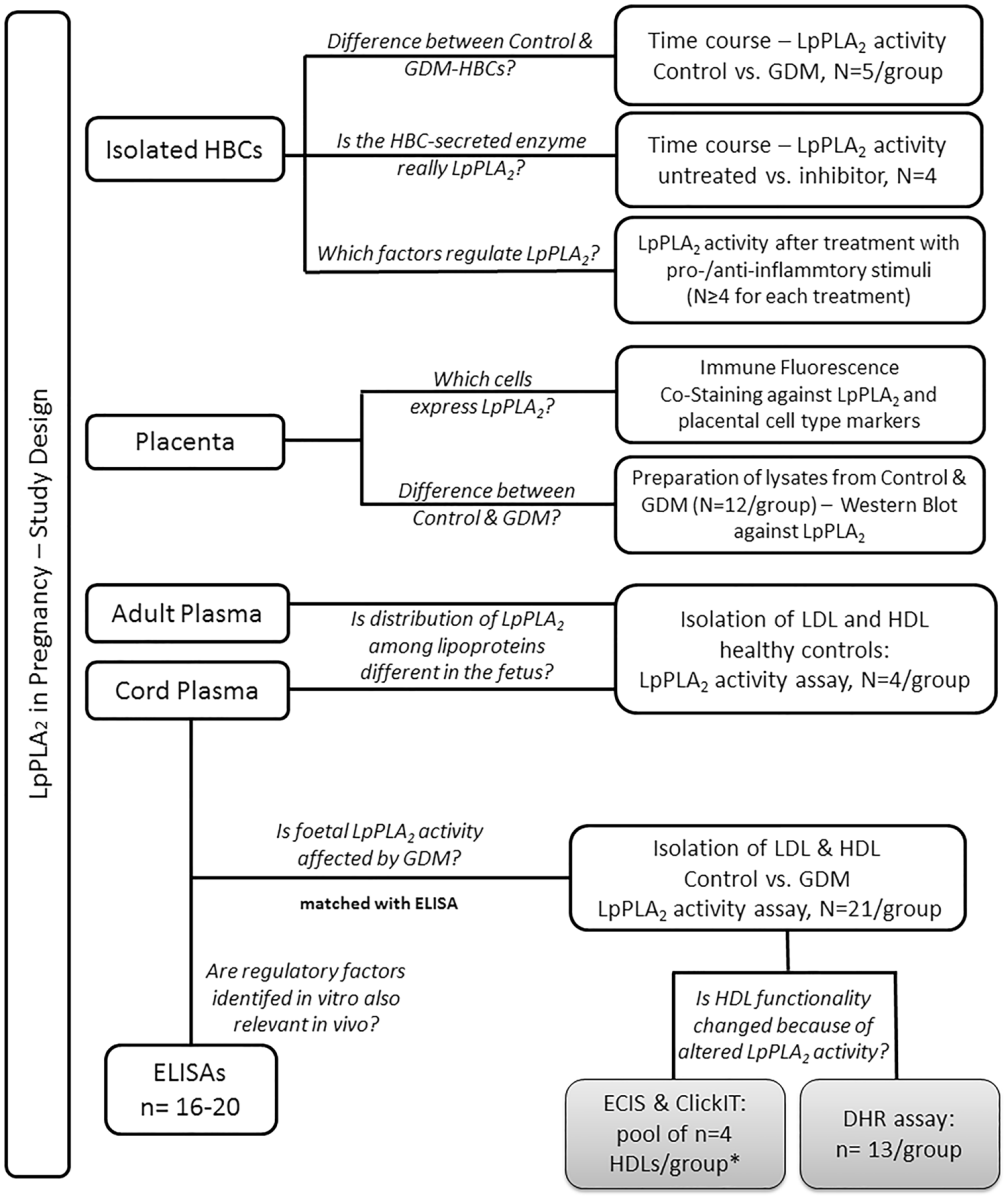
Overview of the study design and experimental set-up. The study investigated LpPLA_2_ in three sample matrices: (1) isolated placental Hofbauer cells, (2) total placental tissue, and (3) on lipoproteins isolated from foetal cord blood plasma drawn immediately after delivery. Tissue and plasma were sampled from both Control and GDM subjects and used as described in the Material and Method section. Abbreviations: HBCs = Hofbauer cells; GDM = gestational diabetes mellitus; LpPLA_2_ = lipoprotein-associated phospholipase A2; LDL = low density lipoprotein; HDL = high density lipoprotein; ELISAs = enzyme-linked immunosorbent assays; ECIS = electrical cell-substrate impedance sensing; DHR = 123-dihydrorhodamine.

Whereas previous studies focused mostly on LpPLA_2_ in maternal circulation, our goal was to investigate LpPLA_2_ in the foetal circulation. We hypothesized that GDM alters LpPLA_2_ levels in placenta and on foetal lipoproteins, which might contribute to changes in lipoprotein functionality. Furthermore, we aimed to broaden the current knowledge about metabolic and inflammatory stimuli regulating LpPLA_2_ activity in macrophages and used human placental macrophages (Hofbauer cells, HBCs), which are of foetal origin, as cell culture model. The study design and experimental set-up are summarized as a flowchart in Fig. 1.

## Results

### LpPLA_2_ is expressed in human placental tissue and secreted by Hofbauer cells

**Figure 2:**
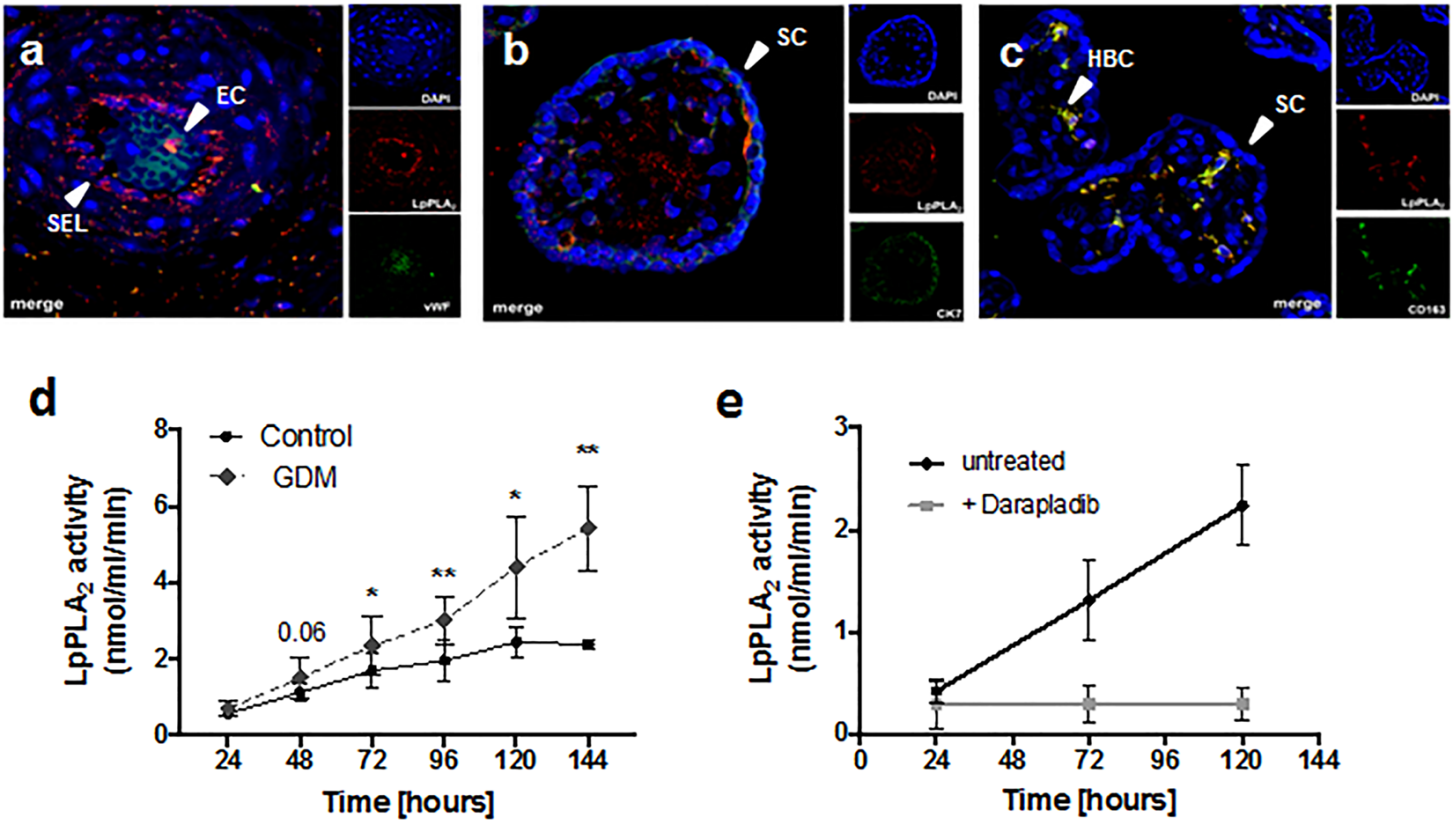
LpPLA_2_ is released by Hofbauer cells and increased in GDM. (**a**–**c**): Immunofluorescence staining of placental tissue. Images are representative of 3 independent experiments (N = 3). (**a**) van Willebrandt factor (vWF, green) was localized to the placental vessel lumen. LpPLA_2_ (red) was localized to villous stroma and sub-endothelial connective tissue layers. EC = endothelial cells, SEL = sub-endothelial layer. **(b)** Trophoblast marker Cytokeratin 7 (CK7, green) was present in the fused syncytial layer of the villous; LpPLA_2_ (red) was localized to villous stroma. SC = Syncytium. (**c**) Co-localization of LpPLA_2_ (red) with CD163 (green), a marker of Hofbauer cells, was observed within villous stroma. HBC = Hofbauer cells. (**d**) LpPLA_2_ activity secreted by HBCs isolated from healthy and GDM placenta (mean ± SD; N = 5 HBC isolations/group; two-way ANOVA). **(e)** LpPLA_2_ activity is abolished by addition of 150 nM Darapladib, a specific LpPLA_2_-inhibitor (mean ± SD, N = 4).

Using immune fluorescence staining of placental tissue sections, LpPLA_2_ protein was localized predominantly in the villous stroma and to some extent also in the sub-endothelial space (Fig. 2a–c). Van Willebrand factor (vWF), a marker of endothelial cells, was expressed along placental vessel linings (Fig. 2a) but vWF did not co-localize with LpPLA_2_ in placental tissue (Fig. 2a). Cytokeratin 7 (CK7), which serves as marker of trophoblast, was localized all around the fused syncytial layer of the villus, but did not co-localize with LpPLA_2_ either (Fig. 2b). However, co-localization of LpPLA_2_ with CD163, a marker of HBCs, was observed in the villous stroma (Fig. 2c), suggesting that HBCs are the main cell type in the placenta producing LpPLA_2_.

To prove that GDM affects LpPLA_2_ production and thereby activity from macrophages, HBCs from control and GDM placental tissue were isolated and cultivated under the same conditions for six days. LpPLA_2_ activity was determined in the collected supernatants for each time point. After 48 h, GDM-HBCs secreted more active LpPLA_2_ than control HBCs, and at 72 h the difference became statistically significant (Fig. 2d) and persisted until day 6 (2.3-fold increase, p = 0.002). To demonstrate that the enzyme activity corresponds specifically to LpPLA_2_, cells were exposed to Darapladib, a selective inhibitor of LpPLA_2_ activity^38^. LpPLA_2_ activity was completely absent (−93%) in the HBCs supernatants after inhibitor treatment (Fig. 2e).

### Insulin and Leptin regulate LpPLA_2_ activity *in vitro*

**Figure 3:**
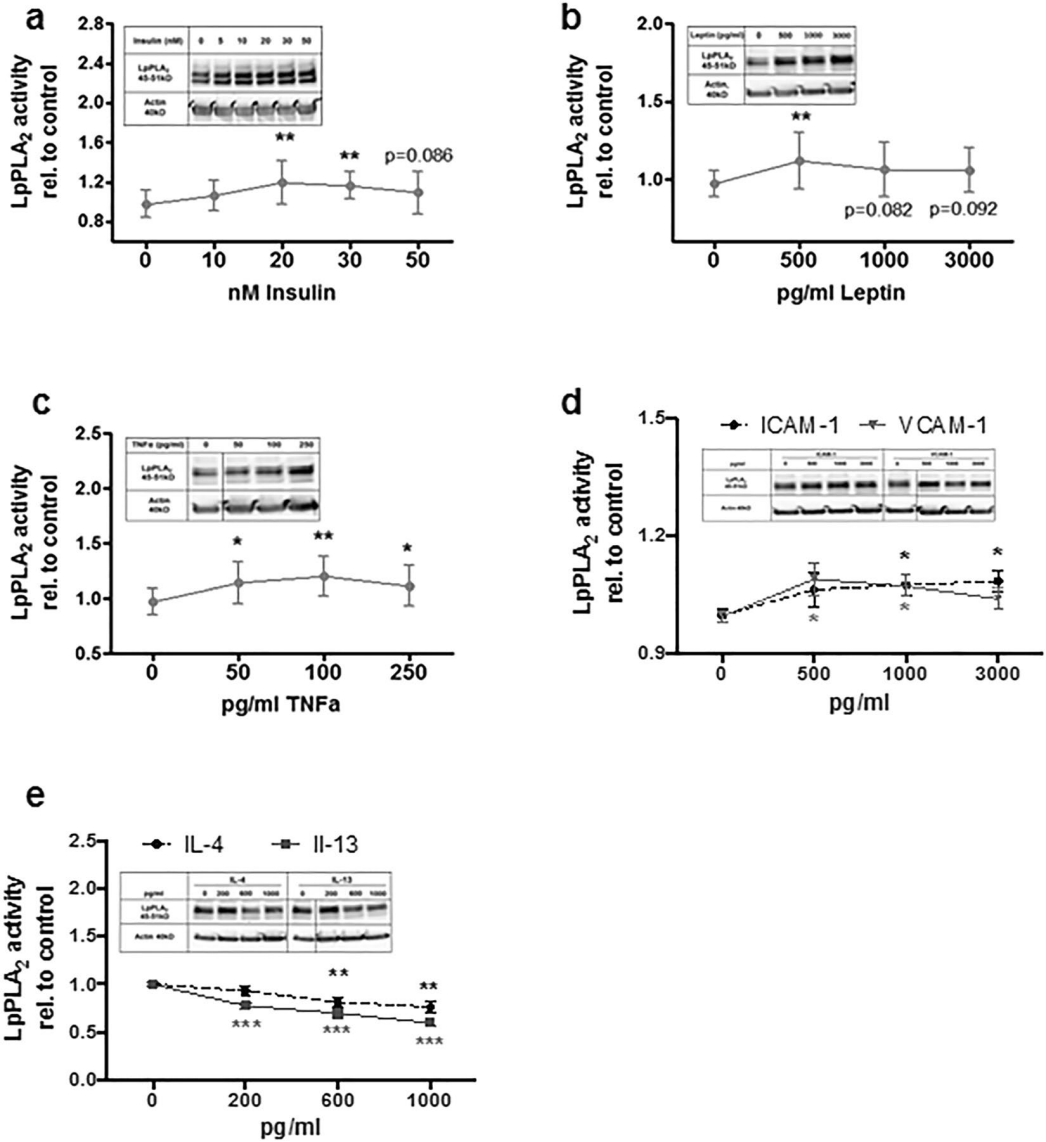
Metabolic hormones, cytokines and adhesion molecules modulate LpPLA_2_ levels in Hofbauer cells. (**a**) Effect of Insulin on LpPLA_2_ protein and activity in HBCs. (**b**) Effect of Leptin on LpPLA_2_ protein and activity in HBCs. (**c**) Effect of TNFα (tumour necrosis factor α) on LpPLA_2_ protein and activity in HBCs. (**d**) Effect of ICAM-1 (intra-cellular adhesion molecule 1; dashed line) and VCAM-1 (vascular adhesion molecule 1; solid line) on LpPLA_2_ protein and activity in HBCs. (**e**) Effect of IL-4 (interleukin 4; dashed line) and IL-13 (interleukin 13; solid line) on LpPLA_2_ protein and activity in HBCs. For each stimulus, at least four independent experiments were performed. Due to inter-individual variability of LpPLA_2_ levels secreted, the unexposed control was used as baseline level and effects on LpPLA_2_ activity where expressed relative to this control. p-values were calculated using one-way ANOVA.

As maternal GDM appeared to affect LpPLA_2_ activity in HBCs, we assessed whether glucose or insulin are contributing factors. HBCs isolated from control placentae were exposed to glucose or insulin for 72 h, each reagent was added three times, once every 24 h. Supernatants were used to assess LpPLA_2_ activity and cells were harvested for Western Blot. Insulin caused an increase in LpPLA_2_ activity (Fig. 3a) with a maximum effect at 20 nM (+22%, p = 0.004) and also LpPLA_2_ protein increased (Fig. 3a, Western Blot insert). Interestingly, glucose levels did not affect LpPLA_2_ activity (data not shown). In addition to glucose, we also stimulated cells with Leptin. Plasma leptin levels are increased in obese mothers and their children, and maternal obesity is a major predisposing factor for development of GDM. Indeed, leptin also led to a moderate yet significant increase in LpPLA_2_ activity (+15%, p = 0.01 at 500 pg/ml; Fig. 3b) and an even more pronounced increase in LpPLA_2_ protein measured by Western Blot (Fig. 3b, insert).

### Pro-inflammatory cytokines and adhesion molecules stimulate LpPLA_2_ activity *in vitro*

Low-grade inflammation and placental endothelial dysfunction are characteristic for GDM pregnancies. Levels of TNFα (inflammation) or ICAM-1 and VCAM-1 (endothelial dysfunction) are classically elevated in these conditions and may therefore alter LpPLA_2_ activity. To test this, HBCs were exposed to a range of concentrations of the respective cytokines for 72 h. The supernatants were used for LpPLA_2_ activity assay and cell lysates for Western Blots. Although the effects were moderate, all three molecules had a positive effect on LpPLA_2_ activity (Fig. 3c,d). TNFα had a maximum effect of 22% increase (p = 0.003, Fig. 3c), whereas both ICAM-1 and VCAM-1 (Fig. 3d) caused an increase around 9% each (p = 0.015 and p = 0.035, respectively). Concomitant increases in LpPLA_2_ protein were observed for all three stimuli (see respective Western Blot inserts.)

### IL-4 and IL-13 negatively regulate LpPLA_2_ activity *in vitro*

To also test possible effects of anti-inflammatory cytokines on LpPLA_2_ activity, HBCs were exposed to either IL-4 or IL-13 for 72 h. Both cytokines significantly reduced LpPLA_2_ activity (Fig. 3e) as well as LpPLA_2_ protein (Fig. 3e, Western Blot insert). IL-4 treatment dose-dependently decreased LpPLA_2_ activity up to 22% (p < 0.01). Even more pronounced reductions (up to −39%, p < 0.001) were observed with IL-13.

### HDL is the main carrier of neonatal plasma LpPLA_2_ activity

**Figure 4:**
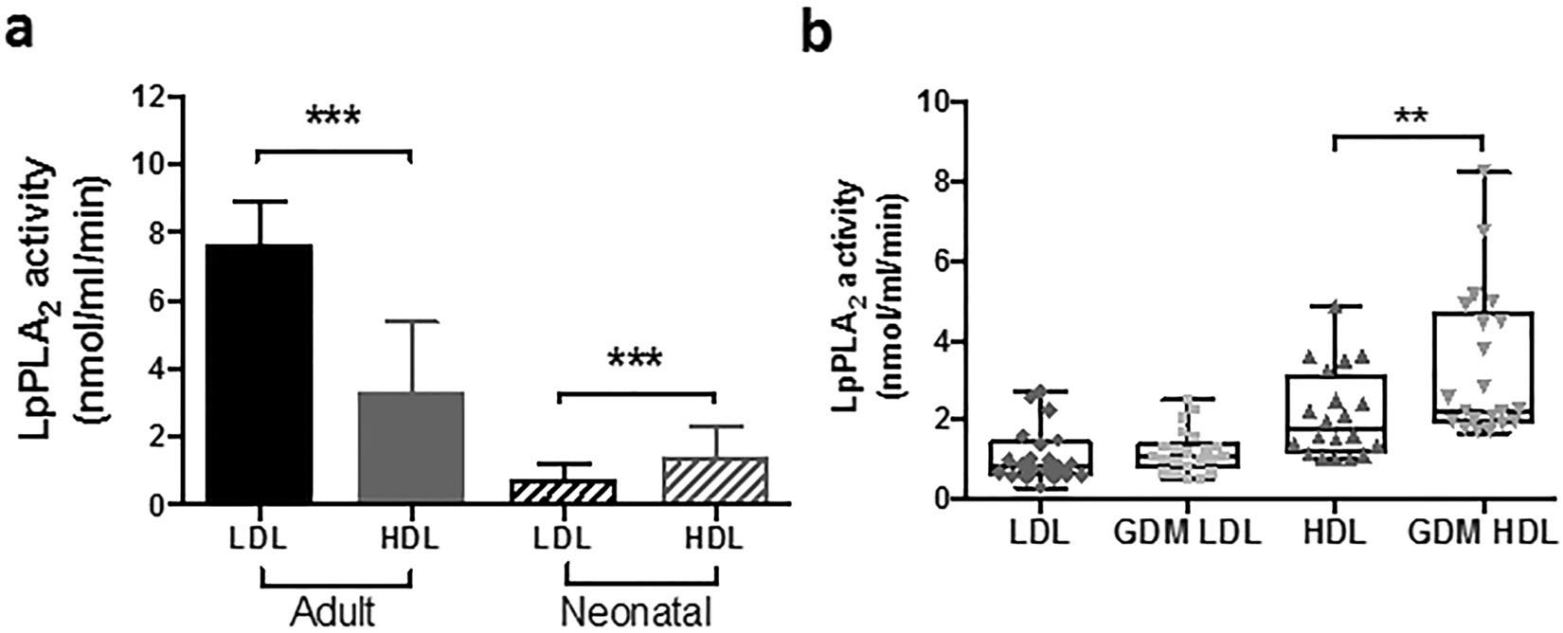
LpPLA2 is present on foetal lipoproteins and increased in GDM. (**a**) Altered distribution of LpPLA_2_ activity among lipoproteins of adult and neonate subjects. Lipoproteins were isolated from adult and cord blood plasma samples (N = 4, mean ± SD, one-way ANOVA). (**b**) LpPLA_2_ activity of foetal lipoproteins from healthy and GDM pregnancies. Data from 3 individual experiments including a total of 21 subjects per group are shown. Statistical significance was calculated using one-way ANOVA. Abbreviations: GDM = gestational diabetes mellitus; LDL = low density lipoprotein; HDL = high density lipoprotein.

In adults, LDL is the main carrier of LpPLA_2_ activity, and its activity is related to the LDL-cholesterol content in adult plasma. In the neonate, however, HDL is the major cholesterol carrying lipoprotein species. For rodent species, were HDL is the main lipoprotein fraction, it has been demonstrated that the majority of LpPLA_2_ activity is associated with HDL^31^. When we compared LpPLA_2_ activity on LDL and HDL particles isolated from healthy adult subjects and foetuses of non-GDM pregnancies, HDL was indeed the main carrier of LpPLA_2_ activity in the foetus (Fig. 4a; 65% activity on HDL vs. 35% on LDL; p < 0.001).

### LpPLA_2_ activity on neonatal HDL is increased by GDM

To assess if GDM causes alterations in LpPLA_2_ activity and distribution in the foetus, LDL and HDL was isolated from cord blood plasma and LpPLA_2_ activity was measured. No differences in the LDL-associated LpPLA_2_ activity was observed between control and GDM foetuses (N = 21/group; Fig. 4b). In contrast, HDL-associated LpPLA_2_ activity was significantly increased in the GDM foetus compared to healthy controls (+54%, p = 0.004, Fig. 4b).

### Insulin and Leptin levels are increased in GDM foetuses ***in vivo***

**Figure 5:**
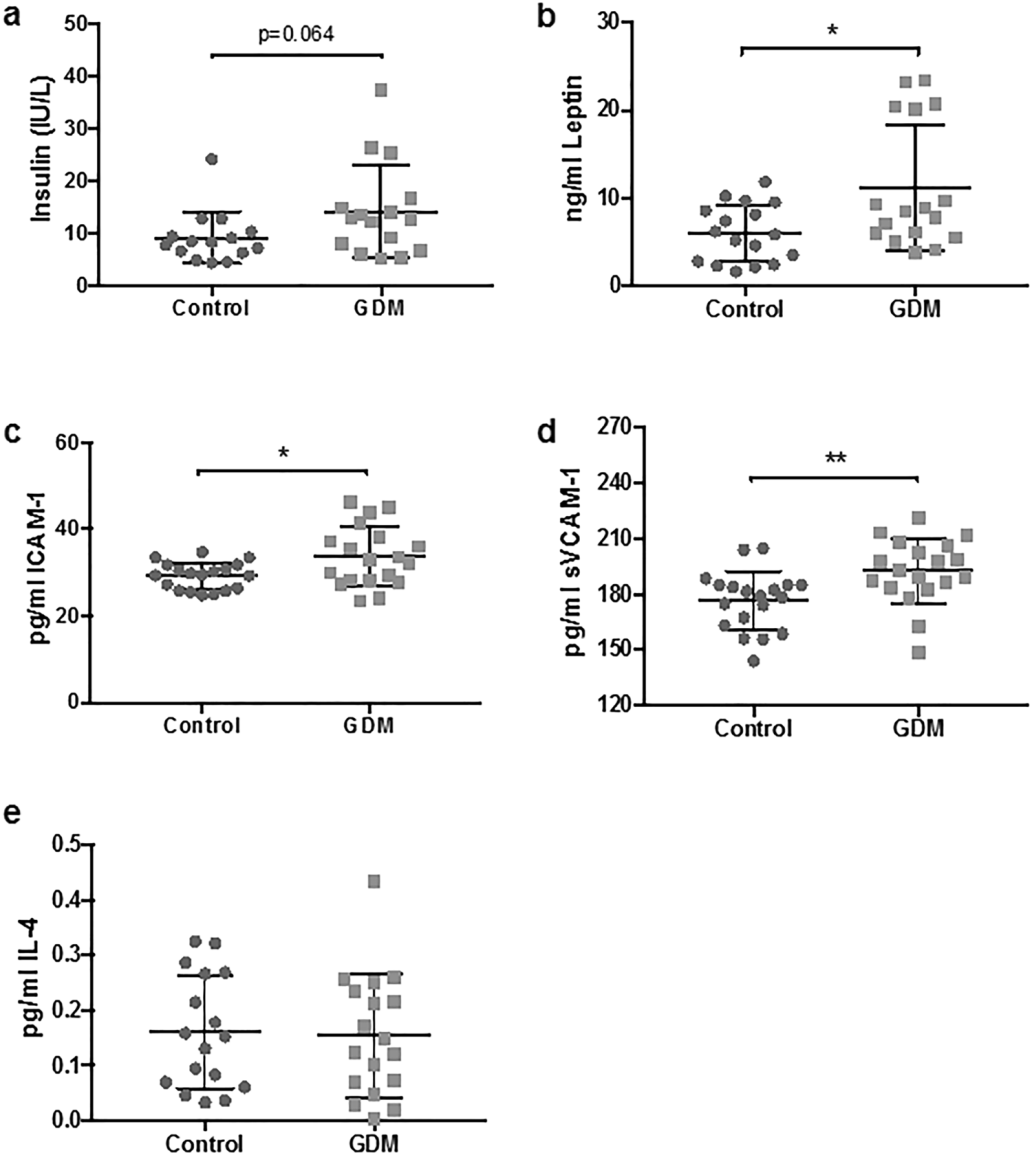
Hormones and Cytokines in neonatal circulation. Enzyme linked immunosorbent assays were used to measure (**a**) insulin, (**b**) leptin, (**c**) intracellular adhesion molecule 1 (ICAM-1), (**d**) vascular adhesion molecule 1 (VCAM-1) and (**e**) interleukin 4 (IL-4). Tumour necrosis factr α (TNFα) and interleukin 13 (IL-13) could not be quantified. All mean ± SD, students t-test.

Given that insulin and leptin increased LpPLA_2_ activity *in vitro*, we assessed whether levels of these hormones are altered in foetal plasma in GDM by ELISA. Plasma insulin (Fig. 5a) and leptin (Fig. 5b) were both increased (+54%, p = 0.06 and +84%, p = 0.01, respectively) in GDM foetuses.

### Pro-inflammatory cytokines and adhesion molecules are increased in foetal plasma

As these pro-inflammatory molecules stimulated LpPLA_2_ activity *in vitro*, TNFα, ICAM-1, and VCAM-1 levels were measured in plasma of control and GDM neonates. TNFα levels were below the assay detection limit in the majority of samples and could not be reliably quantified. ICAM-1 (Fig. 5c) and VCAM-1 (Fig. 5d) levels were significantly increased in GDM foetuses (+14% p = 0.02 and +10% p = 0.006, respectively).

### Anti-inflammatory cytokine plasma levels are unchanged between Control and GDM foetuses

In foetal plasma, IL-4 levels were not different between control and GDM group (Fig. 5e). IL-13 levels were below limit of detection of the ELISA and could not be quantified.

### Foetal HDL-associated LpPLA_2_ activity is correlated with maternal BMI

**Figure 6:**
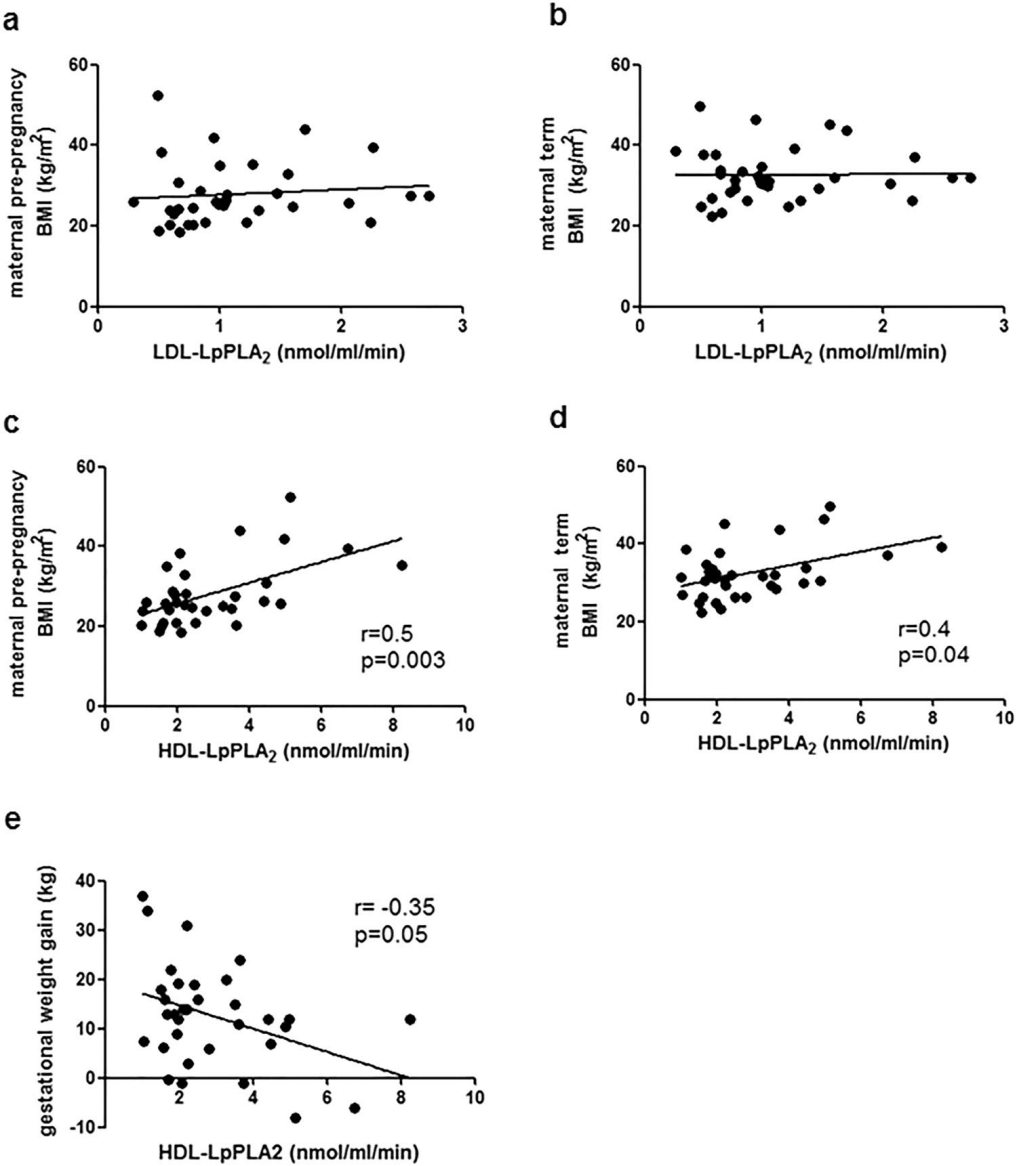
Spearman Correlation of foetal LpPLA_2_ activity with maternal BMI. (**a**) Correlation of LDL-LpPLA_2_ activity in the foetus with maternal BMI. (**b**) Correlation between foetal LDL-LpPLA_2_ activity with maternal BMI at delivery. (**c**) Correlation of HDL-LpPLA_2_ activity in the fetus with maternal BMI before pregnancy. (**d**) Correlation of foetal HDL-LpPLA_2_ activity with maternal BMI at delivery. (**e**) Correlation between foetal HDL-LpPLA_2_ activity with maternal gestational weight gain. Abbreviations: BMI = body-mass index; LDL = low density lipoprotein; HDL = high density lipoprotein; LpPLA_2_ = lipoprotein associated phospholipase A_2_.

As our study cohort for isolation of foetal lipoproteins was not matched for maternal BMI and GDM mothers were significantly overweight compared to control mothers, we tried to investigate maternal BMI as confounding factor, probably affecting foetal LpPLA_2_ activity. Pre-pregnancy BMI in control mothers was average 22.7 ± 3.4 kg/m^2^ vs. 31.5 ± 7.7 kg/m^2^ in GDM women (p < 0.001). At term, BMI in control women was average 28.6 ± 4.0 kg/m^2^, and 34.9 ± 6.2 kg/m^2^ in GDM (p < 0.001). Whereas LDL-LpPLA_2_ was neither associated with maternal pre-pregnancy BMI (Fig. 6a) nor BMI at delivery (Fig. 6b), HDL-LpPLA_2_ showed strong positive correlation with pre-pregnancy BMI (Fig. 6c, r = 0.5, p = 0.003) and moderate correlation with BMI at term (Fig. 6d, r = 0.4, p = 0.04). Overweight mothers are recommended to gain less weight during pregnancy, so gestational weight gain in GDM mothers was smaller compared to controls (average 9.3 ± 9.6 kg in GDM vs. 15.9 ± 9.7 kg in controls, p = 0.05) and as a consequence HDL-LpPLA_2_ activity was inversely correlated with maternal gestational weight gain (Fig. 6e, r = −0.35, p = 0.05).

### LpPLA_2_ is inversely associated with surrogate markers of oxidative stress in placenta and foetal plasma

**Figure 7:**
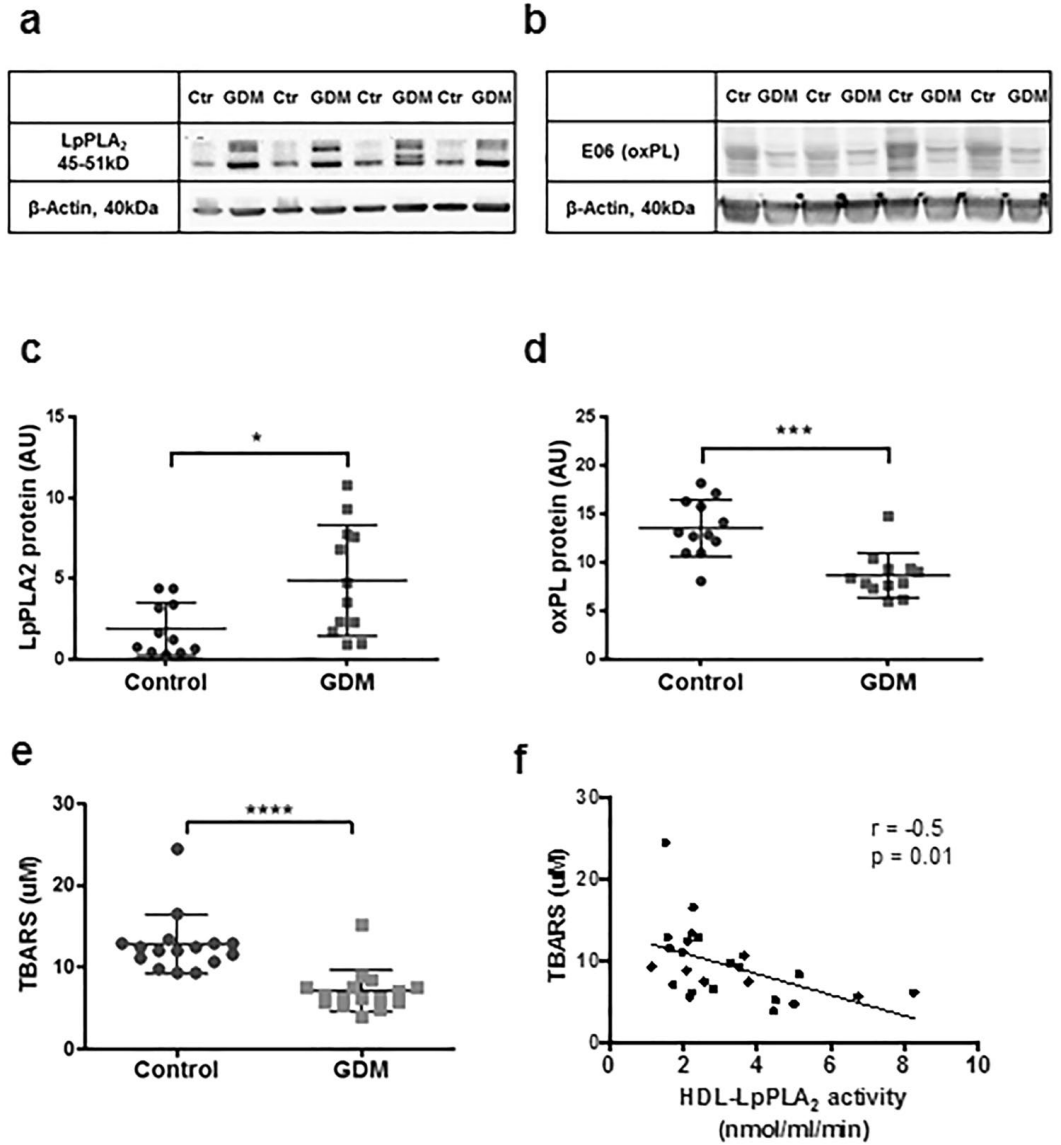
Surrogate markers of oxidative stress are inversely associated with LpPLA_2_ in placenta and foetal circulation. (**a** and **b**) Western Blot of control and GDM placental tissue lysates against LpPLA_2_ (**a**) and oxidized phospholipid residues (E06 oxPL) (**b**). ß-Actin was used for normalization as loading control. One representative out of N ≥ 3 experiments is shown. Images shown have been cropped; uncropped original files are available as Supplementary Information. (**c** and **d**) Densitometric analysis of LpPLA_2_ protein and proteins modified by oxidized phospholipids (oxPL) in control and GDM placentae (N = 12 per group). Data from 3 individual experiments were pooled, mean ± SD, t-test. (**e**) Thiobarbituric acid reactive species (TBARS) were measured as surrogate of oxidative stress in neonatal cord plasma (mean ± SD, N = 16 per group, t-test). (**f**) Pearson correlation of TBARS levels in foetal circulation with HDL-LpPLA_2_.

To test if LpPLA_2_ action might be relevant in situations of placental and foetal oxidative stress, we measured surrogate markers of oxidative stress in placenta and cord blood plasma. In GDM placentae, LpPLA_2_ protein was more abundant than in control placentae (Fig. 7a). This was paralleled by lower levels of oxPL-modified proteins (detected by the E06-oxPC antibody) in GDM placental tissue (Fig. 7b).

LpPLA_2_ is a highly N-glycosylated enzyme, producing multiple bands in Western Blot, ranging in size from 42-51 kDa, depending on the degree of glycosylated residues present^39^. Interestingly, in GDM tissue we detected more than one band, as opposed to control tissue, where only one defined band was detectable. The possible functional implications of the N-glycosylation is not fully clear, but may contribute to directing LpPLA_2_ binding towards HDL instead of LDL^39^. We further investigated the glycosylation of LpPLA_2_ using an enzymatic de-glycosylation kit, but could not find differences in the glycosylation of LpPLA_2_ in GDM placenta and on GDM-HDL with respect to the shift in molecular weight or retention factor after de-glycosylation (data not shown).

Whereas densitometric analysis showed that placental LpPLA_2_ levels were increased in GDM (Fig. 7b, p = 0.04), oxPL protein modification was significantly reduced in GDM placental tissue (Fig. 7c, p < 0.001), suggesting an inverse association between LpPLA_2_ and its substrate; Pearson correlation, however was non-significant (r = −0.36, p = 0.09, data not shown).

Furthermore, we measured the concentrations of thiobarbituric acid reactive substances (TBARS) as index of lipid peroxidation in foetal plasma. They were significantly lower in GDM than in controls (Fig. 7d, p < 0.0001). TBARS levels were inversely correlated with HDL-associated LpPLA_2_ activity (Fig. 7e, r = −0.5, p = 0.01) thus confirming our hypothesis that LpPLA_2_ might act against oxidative stress in placenta and foetus.

### HDL-associated LpPLA_2_ contributes to anti-inflammatory, anti-oxidative functionalities of HDL

**Figure 8:**
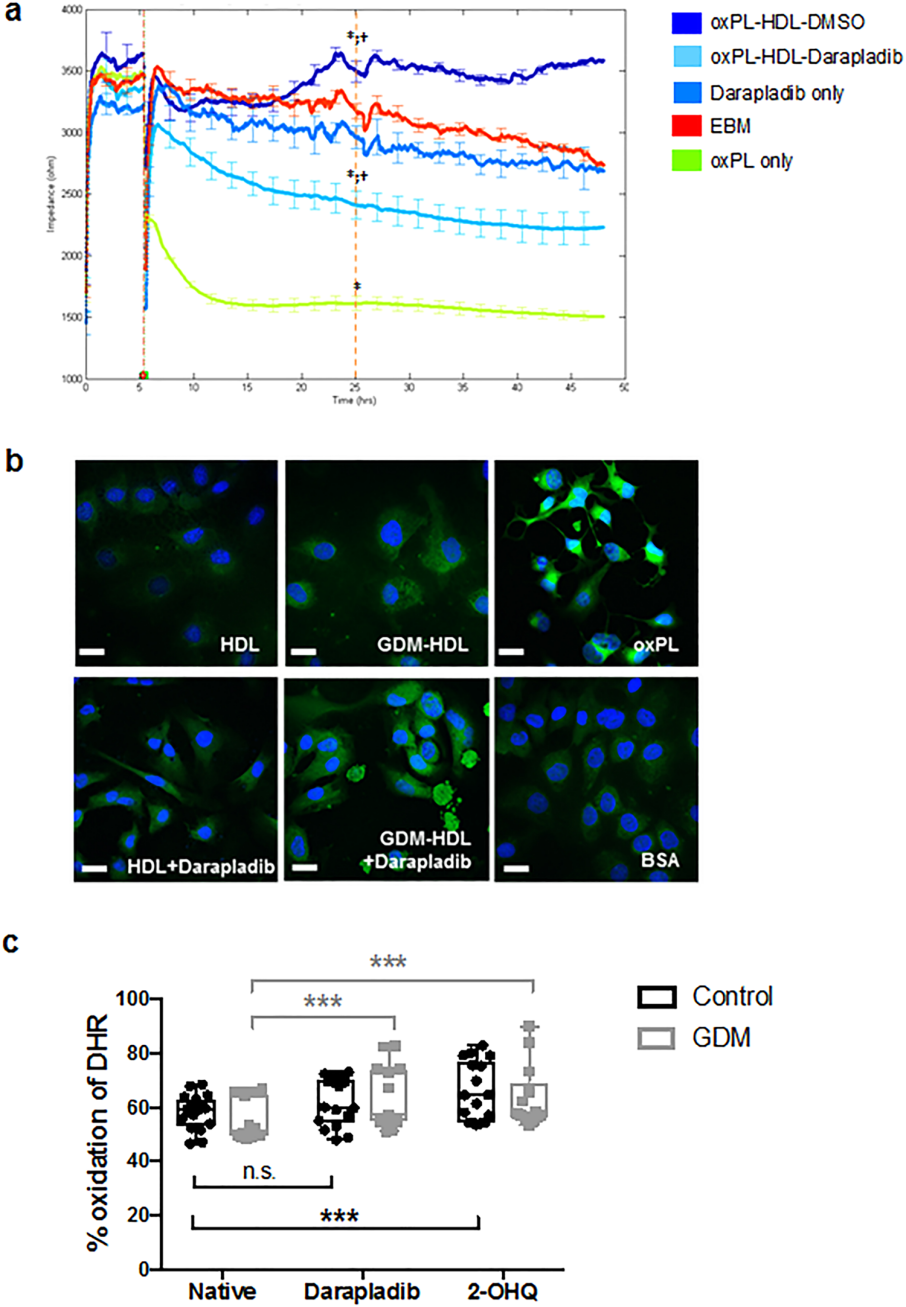
Anti-oxidative potential of HDL-LpPLA_2_ on neonatal placental endothelium. (**a**) Barrier function assay of placental endothelial cells exposed to (i) oxidized phospholipid mix (oxPL, 15 ug/ml, green), (ii) oxPL plus neonatal HDL (15 ug/ml + 200 ug/ml, dark blue) and (iii) oxPL plus neonatal HDL in the presence of Darapladip (15 ug/ml + 200 ug/ml + 150 nM, resp.; turquoise). Darapladib alone (light blue) did not show any off-target effects compared to endothelial basal medium (EBM, red). One out of five representative experiments is shown. *p < 0.05 compared to EBM, †p < 0.005 compared to oxPL. (**b**) Anti-oxidative effects of HDL-LpPLA_2_ in a cell-based assay of lipid peroxidation (ClickIT™ assay). Lipid peroxidation was visualized based on linoleamid alkyl and fluorophores on a laser scanning microscope using defined settings for all pictures taken to make them comparable. (**c**) Cell free assay of Control HDL (black) and GDM- HDL (grey) anti-oxidative potential based on the oxidation of 123-dihydrorhodamine (DHR). In addition to LpPLA_2_-inhibition by Darapladib, also Paraoxanase-1 (PON-1) was inhibited by 2-hydroxyquinolone (2-OHQ). One-way ANOVA was used to test for significance.

The influence of HDL-associated LpPLA_2_ on foetal HDL function was assessed using cell-based and cell-free functional assays. First, real-time paracellular passage between the endothelial cells (the barrier function) was monitored by using electric cell-substrate impedance sensing (ECIS, Fig. 8a). Placental arterial endothelial cells were exposed to oxPL alone, oxPL that had been pre-incubated with native foetal HDL, or foetal HDL treated with Darapladib to inhibit LpPLA_2_ activity. Additionally, to exclude possible off-target effects, cells were also treated with inhibitor alone. As negative control, untreated cells grown in endothelial basal medium (EBM) only were included. Figure 8a shows one representative ECIS experiment. After recording of a 5 h baseline, the respective compounds were added. Both set ups containing HDL protected the cellular barrier integrity compared to oxPL alone (p = 0.005 for oxPL-HDL-DMSO and p < 0.001 for oxPL-HDL-Darapladib at 25 h). However, only when LpPLA_2_ was active on HDL, the impedance increased compared to EBM control (p = 0.04 at 25 h). Inhibition of LpPLA_2_ caused a decrease in barrier function compared to EBM control (p < 0.001 at 25 h). No significant off-target effects of Darapladib were observed. Although a total of five experiments showed comparable results (*Supplemental* Fig. 1), considerable inter-individual variation between the five primary endothelial cell isolations (variance in baseline impedance, more immediate vs. more prolonged response to compound addition, etc.) precluded statistically significant results.

In another cell-based assay measuring lipid peroxidation, we compared native foetal HDL with inhibitor-treated HDL. Placental endothelial cells were grown on slides suited for fluorescence microscopy and incubated with either native HDL, inhibitor-treated HDL, oxPL alone (positive control) or BSA (negative control). Also, native GDM-HDL and inhibitor-treated GDM-HDL were used for incubation. ECs were subsequently exposed to linoleamide alkyne (LAA), which incorporates into cell membranes. Upon lipid peroxidation, it leads to the formation of aldehyde-protein-adducts, which can be detected and visualised by azide-modified fluorophores. This assay allows assessing lipid peroxides within cells. We found that oxPL alone leads to high fluorescence signals reflecting a high degree of lipid peroxidation in the cells, thus corroborating the ECIS results. In addition, oxPL exposure induced a change in morphology, likely due to an increase in apoptosis (Fig. 8b, upper right corner). Between BSA treated (Fig. 8b, lower right corner) and native HDL treated cells (Fig. 8b, upper left corner) no apparent morphological difference was observed, and fluorescence (=lipid peroxidation) in both treatments was much lower than in the oxPL positive control. Finally, in cells exposed to Darapladib-treated HDL (Fig. 8b, lower right corner), there was a higher degree of lipid peroxidation detectable compared to native HDL or BSA control, but clearly lower than in the positive control. For GDM-HDL (upper centre), lipid peroxidation seemed a bit more compared to Control HDL and BSA negative control; however, inhibition of LpPLA_2_ by Darapladib (lower centre), further exacerbated cellular peroxidation, resulting in a similar effect as in Control HDL treated cells.

Furthermore, a cell-free assay using Dihydrorhodamine-123 (DHR) was performed^40^ to compare the anti-oxidative capacity (AOC) of native HDL (both Control and GDM) with HDL treated with Darapladib to inhibit LpPLA_2_. In addition, we also included HDL treated with 2-hydroxyquinolone (2-OHQ) to inhibit Paraoxanase-1 (PON-1), another anti-oxidative enzyme on HDL. Native Control HDL oxidised 58% of DHR. After inhibition of LpPLA_2_ (62% oxidation, n.s., p = 0.085) or PON-1 (66% oxidation, p < 0.001) DHR oxidation was increased (Fig. 8c). GDM-HDL oxidised 55% of DHR, and after treatment with Darapladib oxidation increased to 64% (p < 0.001); inhibition of PON-1 also led to an increase in oxidation up to 64% (p < 0.001, Fig. 8c). These results show that both enzymes, LpPLA_2_ and PON-1, contribute to the anti-oxidative capacity of HDL; however, in GDM-HDL the contribution of LpPLA_2_ to the AOC might be more essential than in control HDL.

## Discussion

Here we report that GDM- and obesity-associated metabolic and inflammatory derangements in pregnant mothers alter LpPLA_2_ activity with functional consequences in the placenta and foetus. Our results suggest that the increased release of LpPLA_2_ from placental HBCs is not caused by hyperglycaemia, but rather by hyperinsulinemia and inflammatory cytokines.

As placental HBCs are of foetal origin^41^ we could corroborate *in vitro* findings with related *in vivo* data of foetal plasma parameters. This study design combining cellular *in vitro* with *in vivo* studies in human is a major strength, and to the best of our knowledge also unique in addressing why and how LpPLA_2_ is altered in the foetus and foeto-placental tissues. Provided that our findings at the end of pregnancy reflect to some extent the later period of pregnancy, LpPLA_2_ activity may have beneficial, protective effects for the developing placenta and foetus.

We found that LpPLA_2_ activity was increased in foeto-placental macrophages from GDM pregnancies, as well as on foetal lipoproteins from an obese GDM cohort. Specifically, HDL-associated LpPLA_2_ activity was increased, which may have functional implications for the HDL particles. Previous studies suggested that LpPLA_2_ might exert pro- and anti-inflammatory activities dependent on its lipoprotein carrier. LDL-associated LpPLA_2_ exerts pro-inflammatory actions, whereas HDL-associated LpPLA_2_ exerts anti-inflammatory actions^6,42,43^. We identified HDL as the major carrier of foetal LpPLA_2_ activity which is in line with other observations demonstrating HDL as the main cholesterol carrying lipoprotein sub-fraction in cord blood^44^, whereas it has been shown in adults that LpPLA_2_ activity correlates strongly with LDL-C and ApoB.

It has to be noted, that a recent study failed to observe increased HDL-associated LpPLA_2_ activity in GDM neonates, nor did the authors find HDL as the major carrier of LpPLA_2_ activity^36^. Differences in the two study designs and methodology may explain this. First, clinical and metabolic parameters of investigated cohorts are substantially different. While Gao and colleagues included only lean GDM mothers, our cohort was biased by a pre-pregnant BMI > 25 kg/m^2^. Second, the investigated foetal cord blood was exclusively collected from the umbilical vein. The umbilical vein transports oxygenated, nutrient-rich blood to the foetal heart. In return, the two umbilical arteries contain deoxygenated and nutrient-poor blood which is transported back to the placenta. Our study, although smaller, used pooled cord blood from vein and arteries and therefore may reflect the systemic foetal environment more closely. Third, LpPLA_2_ activity was assayed by distinct methods; whereas we used a commercially available standardized colorimetric kit, their study used an in-house method employing trichloroacetic acid precipitation in combination with a radioactive tracer. Finally, the LDL-LpPLA_2_ activity was obtained by subtracting the HDL-LpPLA_2_ activity from total plasma activity, whereas our LpPLA_2_ activity was determined in each isolated lipoprotein fraction distinctly.

Overweight and even more so obesity are major pre-disposing factors for the development of diabetes throughout pregnancy^45,46^. Our cohort used for HDL isolation could not be matched for maternal BMI; mothers in the GDM group had significantly higher BMI, both before pregnancy and at time of delivery. Other studies have faced the same problem^36,47^.

We attempted to answer the question if maternal BMI affects LpPLA_2_, and performed correlation analysis of foetal LpPLA_2_ activity with maternal BMI. For LDL-associated LpPLA_2_ activity, making up only the minor portion of foetal total plasma LpPLA_2_, no correlations were found. We did, however, find a strong correlation of HDL-associated LpPLA_2_ activity with maternal pre-pregnancy BMI. Correlation with BMI at time of delivery was more moderate, which can be explained by differential weight gain in the control and GDM group. Also, HDL-LpPLA_2_ activity was inversely correlated with maternal weight gain. Additionally, in samples from a different obese, non-GDM cohort, we also found a correlation between LpPLA_2_ protein expression in placenta and maternal BMI (Schliefsteiner *et al*., unpublished data). This corroborates the notion that our study – as well as others – might be flawed by a considerable (co-)effect of maternal obesity along with maternal diabetes.

Notably, several studies have shown that maternal obesity and GDM do not only impose a risk on maternal health but also affect long term health of their children. Neonates from GDM pregnancies are prone to be macrosomic, have increased fat depositions^48^ and an increased risk for development of Type 2 diabetes later in life^49^. Several studies have investigated LpPLA_2_ levels in obese children and adolescents and all found correlations between LpPLA_2_ activity and BMI^50,51^ as well as fat mass and waist circumference^52^. Other studies also showed that LpPLA_2_ activity is a predictor of Type 2 diabetes development^19^. It is tempting to speculate if increased LpPLA_2_ in GDM children might be causally related to development of obesity and diabetes later on, or if it could at least serve as a risk-predictor. However, more focused prospective studies regarding metabolic variations and follow-up of patients with and without risk factors are needed in order to clarify the role of LpPLA_2_ in these settings.

We also sought to identify *in vitro* molecular regulators of LpPLA_2_ activity on HBCs, which may contribute to the GDM associated changes. Elevated glucose and insulin levels in the cord blood are key features of a GDM pregnancy^15,47^. Foetal insulin is able to regulate placental gene expression^53^ and HBCs are rich in insulin-receptors^54^. Glucose and insulin were therefore obvious candidates for regulating LpPLA_2_ activity. Whereas glucose did not have any effect, insulin increased LpPLA_2_ activity. Leptin as a major adipogenic hormone also increased LpPLA_2_ activity in HBCs and foetal plasma leptin levels were significantly elevated in the obese GDM group. Our findings at the foeto-placental axis are consistent with the role of insulin and leptin as adiposity signals, which are both positively correlated with body weight in general and adipose tissue mass in particular^55^, further indicating that not only GDM but also maternal BMI influences perinatal outcome. Both hormones regulate LpPLA_2_ and may thus account for chronic low-grade inflammation in the GDM placenta^22^ as well as (placental) endothelial dysfunction^56^. Pro-inflammatory TNFα, and to a lesser degree also endothelial adhesion molecules ICAM-1 and VCAM-1, also induced LpPLA_2_ activity in HBCs. TNFα could not be quantified in cord blood plasma, but both ICAM-1 and VCAM-1 levels were increased in the obese GDM group. ICAM-1 and VCAM-1 have been established as circulating markers of endothelial activation^57,58^ and increased plasma levels may also contribute to increased HDL-LpPLA_2_ activity in GDM neonates.

Importantly, anti-inflammatory cytokines such as IL-4 and IL-13 significantly decreased LpPLA_2_ activity from HBCs, suggesting that LpPLA_2_ expression is responsive to the macrophage micro-environment, which would also explain up- and down-regulation of LpPLA_2_ during and after acute-phase response^59^. Similar to our observations, others found that peripheral blood monocytes upon stimulation with IL-4 secrete significantly less LpPLA_2_ compared to cells stimulated with M-CSF (macrophage colony stimulating factor)^60^.

Unlike clinical studies in the past, which linked LpPLA_2_ mass and/or activity with clinical parameters in an associative manner, we aimed to identify molecules causal for regulating LpPLA_2_ activity *in vitro*. However, we did not investigate the signal transduction mechanisms by which these regulators orchestrate LpPLA_2_ activity. One might consider this a limitation of our study. From the limited amount of studies on signalling pathways activating LpPLA_2_^61,62^ and current knowledge about signal transduction pathways activated by pro-inflammatory cytokines^63^ and insulin and leptin^55^, we assume that the regulators of LpPLA_2_ activity identified in our study act through mechanisms dependent on p38 and PI3K within the MAPK pathway.

The functional consequences of altered LpPLA_2_ activity in placenta and foetus may be important for maintaining stress levels low at the foeto-placental interface. GDM and obesity are associated with oxidative stress in the placenta, which is paralleled by higher antioxidant levels^64^. The inverse relationship between LpPLA_2_ protein and oxidized phospholipids in placental tissue suggests that lower oxPL levels could be a result of increased LpPLA_2_ action, which is in line with an anti-oxidative role of LpPLA_2_. In cord blood, TBARS were measured as a surrogate marker of oxidative stress and their levels inversely correlated with HDL-LpPLA_2_ activity. Collectively, these results suggest a local and specifically tight regulation of anti-oxidative defence mechanisms within the human placenta and foetus. Of note, different from our findings, increased TBARS or malondialdehyd levels in cord blood of GDM pregnancies were reported by others^65,66^. However, anti-oxidant enzymes (e.g. superoxide dismutase) were also increased in GDM^65^, so total anti-oxidative potential was unchanged in these studies.

LpPLA_2_ circulating on LDL and HDL is in constant contact with macro- and microvascular endothelium. We therefore considered that LpPLA_2_ might have an effect on endothelial function. Using electrical cell substrate impedance sensing (ECIS), we demonstrated the positive effect of foetal HDL-LpPLA_2_ on placental endothelial barrier function and that this effect was abolished when HDL-LpPLA_2_ activity was inhibited. One might speculate that elevated HDL-LpPLA_2_ in GDM could be a protective counter-mechanism against the endothelial dysfunction commonly observed in GDM^58^. By pre-incubating HDL and LpPLA_2_-inhibited HDL with oxPL, we could also describe an anti-oxidative effect of LpPLA_2_. This points towards a role of LpPLA_2_ as an anti-oxidant, and in the regulation of vascular permeability. Furthermore, a cell free assay demonstrated that HDL-LpPLA_2_, specifically on GDM-HDL, contributes to the total anti-oxidative potential of the HDL particle.

In clinical trials, the specific LpPLA_2_-inhibitor Darapladib offered no benefit for patients^67^. Nevertheless, physicians identified low LpPLA_2_ activity as goal to improve patient health. Our *in vitro* study in human macrophages points towards other treatment options than inhibitors to achieve lower plasma LpPLA_2_ activity, such as lifestyle interventions, i.e. diet and exercise^68^, to lower insulin and leptin as well as LDL levels; also lowering LDL levels by statin therapy will reduce LpPLA_2_^69,70^. In addition, lowering pro-inflammatory cytokines or raising anti-inflammatory cytokines by pharmacological means could improve patient outcome, not only by regulating LpPLA_2_ activity but by addressing inflammation more holistically.

## Material and Methods

### Study population

Clinical characteristics for the GDM study are summarised in Supplementary Tables 1 and 2. All subjects gave written informed consent. The study design had been approved by the ethics board of the Medical University of Graz (24–529 ex 11/12). All women underwent an oral glucose tolerance test (OGTT) between 24 and 28 weeks of gestation. Gestational diabetes was diagnosed according to the guidelines of the American Diabetes Association^71^. All individuals in the GDM group were treated only by diet and lifestyle modifications; none of the patients administered insulin. All experimental methods were performed in accordance with the respective approved study protocols.

### Sample collection and storage

For placenta tissue collection (N = 12/group), the placenta was divided into quadrants and a piece of 5–7 mm diameter was punched from each quadrant, reaching from the maternal to the foetal side. The punched piece was cut in half, so that one half contained the chorionic plate (=foetal side, used exclusively in this study) and the other the basal plate (=maternal side). Tissue was either snap frozen in liquid nitrogen and stored at −80 °C until RNA or protein isolation, or was formalin fixed and embedded into paraffin for immune histological examination.

For collection of neonatal cord blood plasma (N = 21/group), cord blood was obtained as mixed blood (from umbilical arteries and vein) directly after delivery of the placenta and cutting of the umbilical cord. Blood was collected in EDTA plasma tubes and centrifuged for 15 min at 2000× g at 4 °C. Plasma was carefully aliquoted and aliquots were stored at −80 °C until further use for ELISAs or lipoprotein isolation. Adult EDTA plasma for isolation of adult LDL and HDL was obtained from healthy female donors (N = 4) at child-bearing age from the blood bank at the General Hospital of Graz and was stored and processed like foetal plasma.

For Hofbauer cell isolation, the placenta was obtained and used as described below.

### Hofbauer cell (HBC) isolation

Both placentae from caesarean section and vaginal delivery were used within 20 min after delivery (N = 13/Control group, N = 5/GDM group). Maternal membranes (decidua) were removed to avoid contamination with decidual HBCss. Tissue was dissected, finely minced and stored overnight in PBS. The next day, 60–100 g tissue was digested in two steps, employing trypsin, and thereafter collagenase A. After digestion, cells were applied onto a percoll gradient to separate cell populations. HBCs appear as band between the 30–35% percoll layers. They were aspirated from the gradient and purified by negative immune selection using antibodies against CD10 and EGFR. Cells were counted and plated in HBCs medium (MaM, ScienCell) supplemented with 5% lipoprotein-deficient serum (LPDS) at a density of 1 × 10^6^ cells/ml. After five days, quality of the primary cells was controlled by immune cytochemistry, using CD163 as a marker for HBCs.

### Time-course experiments

HBCs isolated from control and diabetic placentae (N = 5/group) were cultured up to 6 days and the secreted LpPLA_2_ activity in supernatant was monitored every 24 h. Additionally, control HBCs (N = 4) were also treated for 1, 3, or 5 days with a specific inhibitor of LpPLA_2_ activity, Darapladib (Medchem Express), at a final concentration of 250 nM, and LpPLA_2_ activity was measured. All experiments were carried out using three technical replicates per condition.

### Exposure of HBCs to diabetic environment

For all treatments HBCs were seeded at a density of 1 × 10^6^ cells/ml in 6-well plates; all treatments were performed in triplicates. An untreated control was included in every experiment. *Glucose treatment:* HBCs (N = 4) were exposed to 5, 15, and 25 mM of D-glucose for 72 hours, glucose was added daily. Equimolar controls with L-glucose were included. *Insulin treatment*: HBCs (N = 5) were exposed to 5, 10, 20, 30, and 50 nM of insulin daily for 72 h. *Cytokine treatments*: HBCs were exposed to TNFα (50, 100 and 250 pg/ml; N = 5), IL-4 and IL-13 (200, 600 and 1000 pg/ml, respectively; N = 4), and adhesion molecules ICAM-1 and VCAM-1 (500, 1000 and 3000 pg/ml, respectively; N = 5) each day for 72 hours.

After all treatments, supernatants were collected for activity assay; cells were washed twice with 1x HBSS and lysed using RIPA buffer supplemented with proteinase inhibitor cocktail. Cell lysates were incubated for 30 min on ice, centrifuged at 16000 g for 20 min and stored at −20 °C. Protein concentration of lysates was determined using Bichononic acid (BCA) method (Pierce) following the instruction manual.

### Placental tissue protein isolation

For tissue lysates about one gram of Control or GDM placental tissue (N = 12/group) was homogenized in 2 ml of RIPA buffer with proteinase inhibitor cocktail using an ultra-turrax device. Lysates were centrifuged and supernatant was used to measure protein concentration using the BCA method.

### Western Blot

10 µg of total tissue protein were subjected to PAGE on 4–20% Bis acrylamide precast gels and protein was transferred onto nitrocellulose membranes. Membranes were probed against a polyclonal anti-PAF-AH antibody (Cayman Chemical) recognizing specifically the C-terminal region of LpPLA_2_ and against oxPL using the E06-oxPC antibody (Avanti Polar Lipids). Anti-rabbit and anti-mouse IgG-HRP, respectively, was used to detect protein, ECL substrate for chemiluminescence and a Biorad LAS-400 camera. Protein signal was normalized against β-actin as loading control. To compare between blots, an internal control prepared from THP-1 macrophages was included in every experiment. Densitometric analysis was performed using DigiDoc1000 software (Alpha Innotech). The anti-PAF-AH antibody yields more than one band due to glycosylation of the enzyme, the E06 antibody yields more than one band because it recognizes PC-modified proteins via a phosphocholine headgroup of oxidized phospholipids. For densitometric analysis, the sum of these bands was considered.

### Immune fluorescence staining

Placental tissue sections of 4–6 um thickness were prepared from paraffin-embedded blocks and mounted onto glass slides. Sections were de-paraffinised in xylene and rehydrated in an ethanol dilution series. Antigen retrieval was omitted to not destroy placental villus structure. Sections were blocked using 3% BSA in TBE buffer. Antibodies were mixed in Antibody Diluent and Background Reducing Component (both Dako). Sections were incubated with primary antibody over-night and secondary antibody in the dark for 2 h, respectively and washed several times in between steps. Coverslips were mounted onto glass slides using Prolong Gold Antifade Reagent with DAPI (LifeTechnologies), sealed and stored in the dark at 4 °C. Pictures of sections were taken with a Zeiss LSM510, AxioVert200M microscope.

### Lipoprotein isolation from cord blood

Density of foetal cord blood plasma (8 ml volume) from control and GDM pregnancies (N = 21/group) was adjusted to ρ = 1.24 g/ml using potassium bromide. Plasma was transferred to ultracentrifuge tubes and potassium bromide solution of ρ = 1.006 g/ml was layered on top. Samples were centrifuged at 90.000 g for 4 h at 15 °C in a table top ultra-centrifuge. The LDL layer on the top was collected, the interphase was discarded, and HDL floating in the centre layer of the tube was collected. Lipoproteins were stored at 4 °C, light-protected and under a layer of argon gas to prevent oxidation. Each sample was concentrated to 1.5 ml volume using Vivaspin tubes (MwCo 5 kD, Satorius) and excess potassium bromide was removed using PD10 resin columns. Quality of HDL was assessed measuring total protein and cholesterol, calculating a Protein/Cholesterol ratio (>2:1 were used).

### LpPLA_2_ activity assay

Enzymatic activity of LpPLA_2_ in cell culture supernatant collected from HBCs, as well as on isolated foetal lipoproteins, was measured using a commercially available PAF-AH activity kit (Cayman Chemical). The assay was carried out according to the manufacturer’s instructions and activity was calculated as suggested by the manufacturer and expressed as nmol/ml/min.

### Enzyme linked immunosorbent assays

All ELISAs, IL-4 and ICAM-1 (both Peprotech), IL-13 and VCAM-1 (both RnD Systems), Leptin (Millipore), for foetal cord blood plasma were carried out according to manufacturer’s instructions. Insulin levels were measured by an automated ELISA (Advia Centaur, Siemens).

### Thiobarbituretic acid reactive substances assay

Oxidative status in foetal plasma was measured by surrogate markers of lipid peroxidation. Thiobarbituretic acid reactive substances (TBARS) assay kit (Cayman Chemical), was carried out according to the manufacturer’s instructions.

### DHR-based assay of HDL anti-oxidative function

To assess individual contribution of HDL anti-oxidative enzymes LpPLA_2_ and PON1, foetal HDL was used either in its native form or pre-incubated for 1 h at 37 °C with either Darapladib (250 nM) or 2-hydroxy-quinoline (400 uM). HDL samples (10ug total) were added to 384-well plates with 15 μl of 50 μmol/L Dihydrorhodamine 123 reagent containing 1 mmol/L 2,2′-azobis-2-methyl-propanimidamide-dihydrochloride. The increase in fluorescence (538 nm) per minute was determined over 30 minutes.

### ClickIT Lipid Peroxidation assay

HDL anti-oxidative function by LpPLA_2_ was assessed in a cell based assay (ClickIT™ Lipid Peroxidation Detection Kit, Life Technologies). Placental endothelial cells were isolated as previously described^72^ and grown on chamber slides to 80% confluence. Foetal HDL was used in its native form or pre-incubated with 250 nM Darapladib. Cells were exposed to HDL (200 ug/ml) with or without inhibitor for 2 h at 37 °C. Cells incubated with oxPL (15 ug/ml) or BSA (200 ug/ml) served as positive and negative control, respectively. Subsequently, cells were incubated with LAA for 2 h. For cell fixation, permabilisation and visualisation by Alexa488 fluorophores, the kit was carried out according to manufacturer’s instructions. Laser scanning microscopy (LSM510 AxioVert200M, Zeiss) was used to detect peroxidation-induced fluorescence in the cells.

### Electrical cell-substrate impedance sensing (ECIS)

Human placental arterial endothelial cells were plated in Endothelial Basal Medium (EBM, Lonza) onto chamber slides suited for barrier function measurement (8W10E + PET, ibidi). On an ECIS Z instrument (Applied BioPhysics), baseline was recorded at 4000 Hz for 4 to 6 hours. A mixture of oxPL (15 ug/ml) plus foetal HDL (200 ug/ml) plus either Darapladib (250 nM) or DMSO (vehicle control) – which had been pre-incubated for 1 h at 37 °C – was added to cells. Additionally, cells were exposed to oxPL only, Darapladib only, and EBM only (untreated control). Impedance was monitored over 40 h at 4000 Hz. Analysis of experiments was done using ECIS software (Applied BioPhysics).

### Statistical analysis

All statistics were calculated and graphs prepared using GraphPad Prism v7.0. Where applicable, either two-tailed t-test, one-way or two-way ANOVA were performed, depending on whether two or more groups were compared, respectively. Normal-distribution was tested by Shapiro-Wilks test. If normal distribution failed, non-parametric tests were performed, either Mann-Whitney rank sum or ANOVA on ranks with Dunns test post hoc testing. All data are presented as mean ± SD in tables, bar charts and dot plots. Spearman Correlation was performed for correlation analysis. P-values < 0.05 were considered statistically significant.

## Electronic supplementary material

Supplementary Material
