# Supplementary material for: Maternal Gestational Diabetes Mellitus increases placental and foetal lipoprotein-associated Phospholipase A2 which might exert protective functions against oxidative stress
Source: Sci Rep. 2017 Oct 3;7:12628. doi: 10.1038/s41598-017-13051-6 (PMC5626711; doi:10.1038/s41598-017-13051-6)
Supplement: Supplementary file 1 — Supplementary Material [file 41598_2017_13051_MOESM1_ESM.doc]

**Supplementary Material**

for

**Maternal Gestational Diabetes Mellitus increases placental and foetal lipoprotein-associated Phospholipase A_2_ which might exert protective functions against oxidative stress**

Authors and Affiliations

Carolin Schliefsteiner^1^, Birgit Hirschmugl^1^, Susanne Kopp^1^, Sanja Curcic^2^, Eva Maria Bernhart^3^, Gunther Marsche^2^, Uwe Lang^1^, Gernot Desoye^1^, Christian Wadsack^1,*^

1. Dept. of Obstetrics and Gynaecology, Medical University of Graz, Austria
2. Dept. of Clinical and Experimental Pharmacology, Medical University of Graz, Austria
3. Inst. of Molecular Biology and Biochemistry, Medical University of Graz, Austria

- Please address correspondence to: [christian.wadsack@medunigraz.at](mailto:christian.wadsack@medunigraz.at)

**Supplementary Figures**

Supplementary Figure S1 **Impedance change (delta impedance) of barrier function after addition of HDL and Darapladib-treated HDL.** Data from five experiments with five different primary endothelial cell isolations (mean±SD, N=5) is depicted. Though not significant, the effects as described in Fig.8a on individual basis were also observed in the data summary. High inter-individual variability, which is typical for primary cells, in the ECs caused high SD, losing statistical power. Abbreviations: oxPL = oxidized phospholipid mix; HDL = high density lipoprotein; EBM = endothelial basal medium; DMSO (vehicle) = dimethylsulfoxide.

Supplementary Tables

|  | **Control group (n=13)** | **GDM group (n=5)** | **p-value** |
| --- | --- | --- | --- |
| Maternal pre-gravid BMI (kg/m^2^) | 23.3±3.11 | 28.6±9.1 | * 0.049 |
| Maternal BMI (kg/m^2^) at term | 28.1±4.0 | 32.0±7.7 | n.s. |
| Gestational weight gain (kg) | 12.4±5.6 | 8.6±5.6 | n.s. |
| Maternal age (years) | 32.0±6.6 | 34.7±2.8 | n.s. |
| Gestational age (weeks) | 39 (IQR: 38.75 to 39.25) | 38 (IQR: 37.0-38.5) | n.s. |
| Mode of delivery | CS 8, SP 5 | CS 4, SP 1 | n.s. |
| Placental weight (g) | 586.2±123.7 | 612.0±161.6 | n.s. |
| Foetal PI (kg/m^3^) | 2.6±0.2 | 2.6±0.3 | n.s. |
| Foetal sex (female ♀/male ♂) | ♀8 ♂5 | ♀1 ♂4 | n.s. |

**Supplementary Table 1 Characteristics of patients whose placentae were used for Hofbauer cell isolation.** Abbreviations: BMI=body-mass index; CS = caesarean section; SP = spontaneous vaginal delivery; PI = foetal ponderal index. Foetal PI is comparable to BMI in adult subjects, but relates weight to the cubed bodylength rather than the squared. This represents the foetal anatomy, with a big head in proportion to the rest of the body, better. All data are presented as mean±SD, except for gestational age for which median and inter-quartile range (IQR) was considered more appropriate.

|  | **Control (n=21)** | **GDM (n=21)** | **p-value** |
| --- | --- | --- | --- |
| Maternal pre-gravid BMI (kg/m^2^)* | 22.7±3.4 | 31.5±7.7 | <0.001 |
| Maternal BMI at term (kg/m^2^) | 28.6±4.0 | 34.9±6.2 | <0.001 |
| Gestational weight gain (kg)* | 15.9±9.7 | 9.3±9.6 | 0.05 |
| Maternal age (years) | 32.8±6.3 | 32.6±5.5 | n.s. |
| Gestational age at delivery (weeks) | 39 (IQR: 38.0-39.25) | 38 (IQR:38.0-39.0) | n.s. |
| Mode of Delivery | CS18, SP3 | CS18, SP3 | n.s. |
| Placental weight (g)* | 672.3±218.8 | 737.8±169.2 | n.s. |
| Foetal PI (kg/m^3^) | 2.6±0.3 | 2.6±0.2 | n.s. |
| Foetal Sex (female ♀/male ♂) | ♀11 ♂10 | ♀10 ♂11 | n.s. |

**Supplementary Table 2 Characteristics of subjects whose cord blood was used for HDL isolation.** Abbreviations: BMI=body-mass index; CS = caesarean section; SP = spontaneous vaginal delivery; PI = foetal ponderal index. Foetal PI is comparable to BMI in adult subjects, but relates weight to the cubed bodylength rather than the squared. This represents the foetal anatomy, with a big head in proportion to the rest of the body, better. *Data on maternal pre-gravid BMI was not available from three subjects in each group. Consequently, gestational weight gain could not be calculated for those patients (N=18/group). For the correlation analysis in Figure 6, only those patients were included were both pre-gravid and term BMI were available. **Placental weight was missing from one subject in each group (N=20/group). All data are presented as mean±SD, except for gestational age for which median and interquartile range was considered more appropriate.

**Uncropped Original Western Blots**


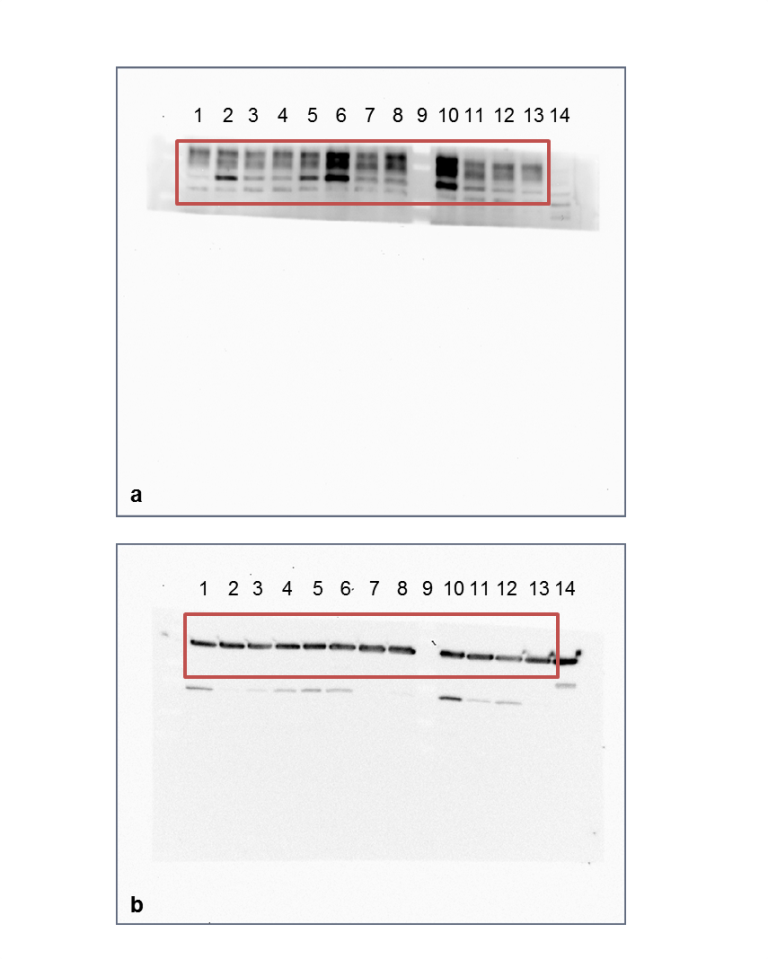


**Supplementary Information 1.** Uncropped Western Blots used for Figure 2. Blot against LpPLA_2_ (a) and corresponding β-Actin (b). Lanes from 1 to 14: 1. Clev050, lean; 2. Clev049, lean; 3. Clev048, lean; 4. Clev155, Grp 2; 5. Clev193, Grp 2; 6. Clev210, Grp 2; 7. Clev059, Grp 3; 8. Page Ruler (Marker); 9. Clev087, Grp 3; 10. Clev107, Grp 3; 11. Clev217, Grp 4; 12. Clev230, Grp 4; 13. Clev238, Grp 4; 14. THP-1 lysate. The red frame indicates the cropped area. The membrane was cut between 55 and 70kD to also incubate with other, bigger proteins of interest (not part of the manuscript). Additionally, for visualisation of LpPLA2 in placental tissue the membrane was covered below 30kD due to an intense unspecific band around 25kDa.


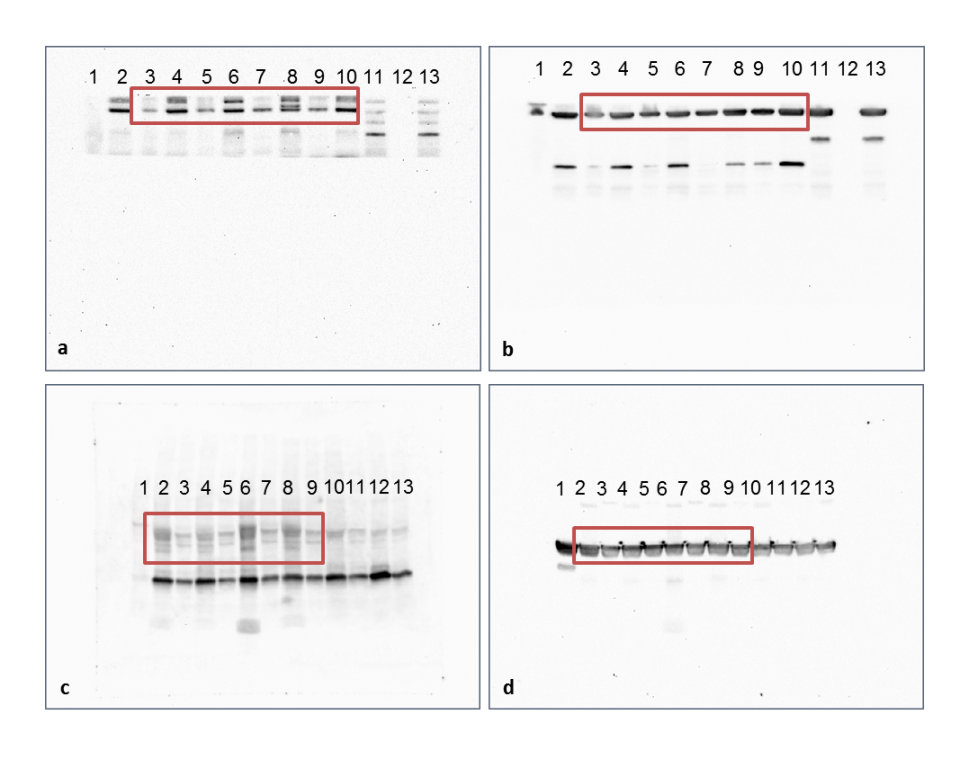


**Supplementary Information 2 Uncropped Western Blots used for Figure 3. (a and b)** Blot against LpPLA_2_ and corresponding β-Actin. Lanes from 1 to 13: 1. Ctr 1; 2. GDM 1; 3. Ctr 2; 4. GDM 2; 5. Ctr 3; 6. GDM 3; 7. Ctr 4; 8. GDM 4; 9. Ctr 5; 10. GDM 5; 11. THP-1 (internal control); 12. Page Ruler (marker); 13. THP-1 (internal control). **(c and d)** Blot against oxPL and corresponding β-Actin. Lanes from 1 to 13: 1. THP-1 (internal control); 2. Ctr 1; 3. GDM 1; 4. Ctr 2; 5. GDM 2; 6. Ctr 3; 7. GDM 3; 8. Ctr 4; 9. GDM 4; 10. Ctr 5; 11. GDM 5; 12. Ctr 6; 13. GDM 6. The red frame indicates the cropped area. The membrane was cut between 55 and 70kD to also incubate with other, bigger proteins of interest (not part of the manuscript). Additionally, for visualisation of LpPLA2 in placental tissue the membrane was covered below 30kD due to an intense unspecific band around 25kDa.


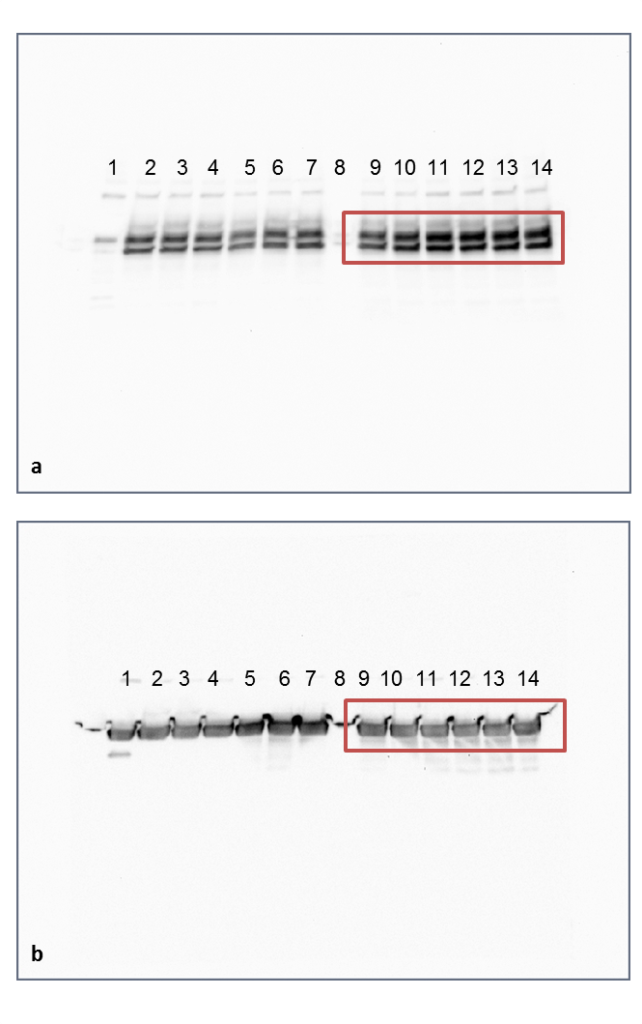


**Supplementary Information 3 Uncropped Western Blots used for Supplementary Figure 2a.** Blot against LpPLA_2_ (a) and corresponding β-Actin (b). Lanes from 1 to 14: 1. THP-1 (internal control); 2. Untreated Control; 3. 5nM Insulin; 4. 10nM Insulin; 5. 20nM Insulin; 6. 30nM Insulin; 7. 50nM Insulin; 8. Page Ruler (marker); 9. Untr. Control; 10. 5nM Insulin; 11. 10nM Insulin;12. 20nM Insulin; 13. 30nM Insulin; 14. 50nM Insulin. The red frame indicates the cropped area.


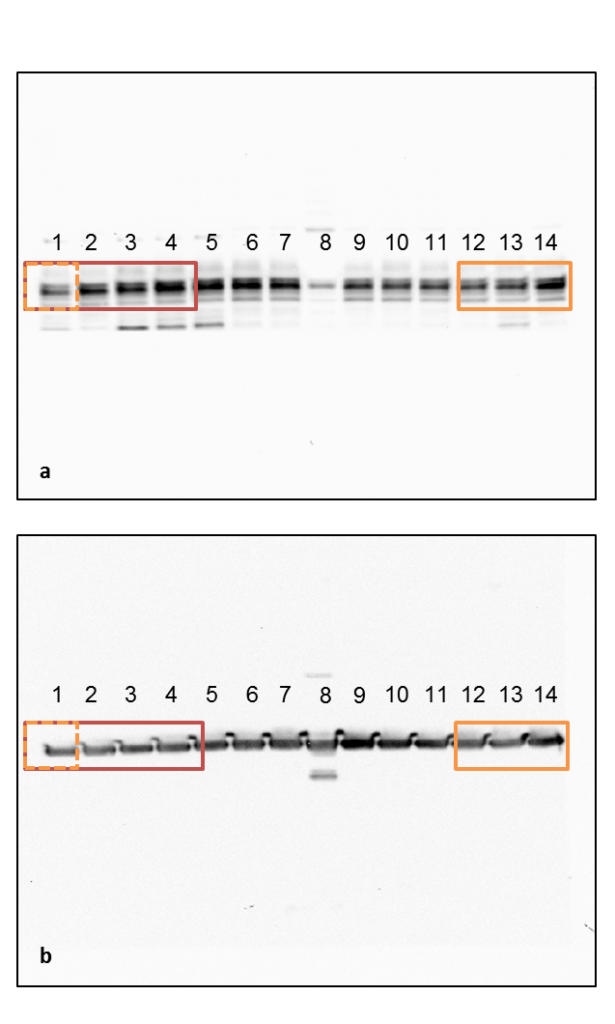


**Supplementary Information 4 Uncropped Western Blots used for Supplementary Figure 2b and 2c.** Blot against LpPLA_2_ **(a)** and corresponding β-Actin **(b).** Lanes from 1 to 14: 1. Untreated Control, 72h; 2. 500pg/ml Leptin; 3. 1000pg/ml Leptin; 4. 3000pg/ml Leptin; 5. 500pg/ml Leptin; 6. 1000pg/ml Leptin; 7. 3000pg/ml Leptin; 8. THP-1 (internal control); 9. 50pg/ml TNFα; 10. 100pg/ml TNFα; 11. 250pg/ml TNFα; 12. 50pg/ml TNFα; 13. 100pg/ml TNFα; 14. 250pg/ml TNFα. The red frame indicates the cropped area for Leptin (Supp. Fig. S2b), the orange frame indicates the cropped area for TNFα (Supp. Fig. S2c), red-orange dashed frame indicates the shared untreated control on this membrane.


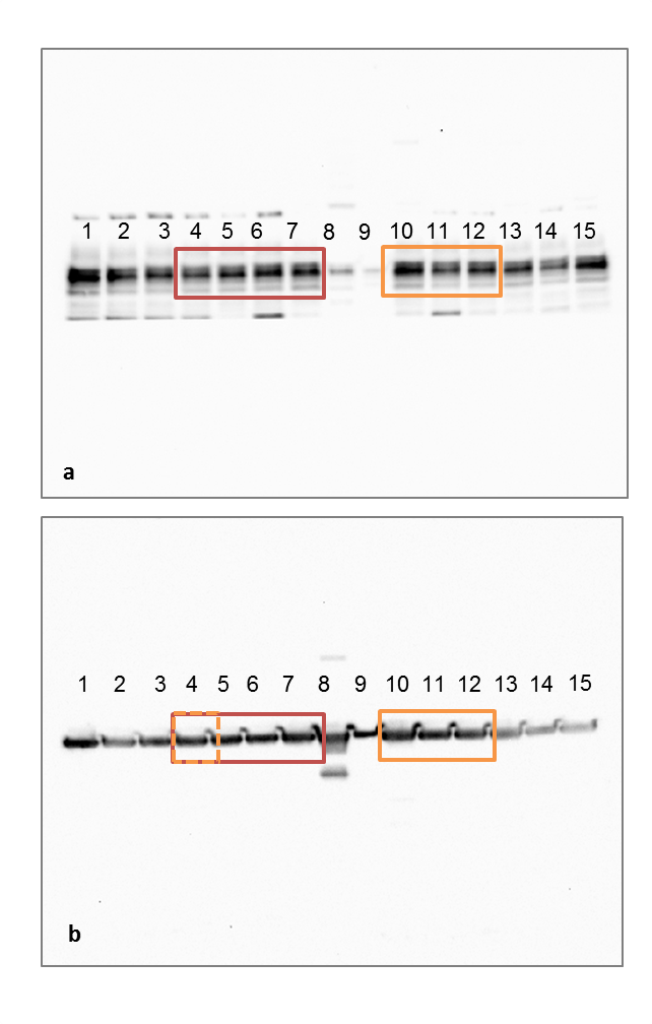


**Supplementary Information 5 Uncropped Western Blots used for Supplementary Figure 2d.** Blot against LpPLA_2_ **(a)** and corresponding β-Actin **(b).** Lanes from 1 to 15: 1. 500pg/ml ICAM-1; 2. 1000pg/ml ICAM-1; 3. 3000pg/ml ICAM-1; 4. Untreated control; 5. 500pg/ml ICAM-1, 6. 1000pg/ml ICAM-1, 7. 3000pg/ml ICAM-1; 8. THP-1 (internal control); 9. Page Ruler (marker); 10. 500pg/ml VCAM-1; 11. 1000pg/ml VCAM-1; 12. 3000pg/ml VCAM-1; 13. 500pg/ml VCAM-1; 14. 1000pg/ml VCAM-1; 15. 3000pg/ml VCAM-1. The red frame indicates the cropped area for ICAM-1, the orange frame indicates the cropped area for VCAM-1, red-orange dashed frame indicates the shared untreated control on this membrane.


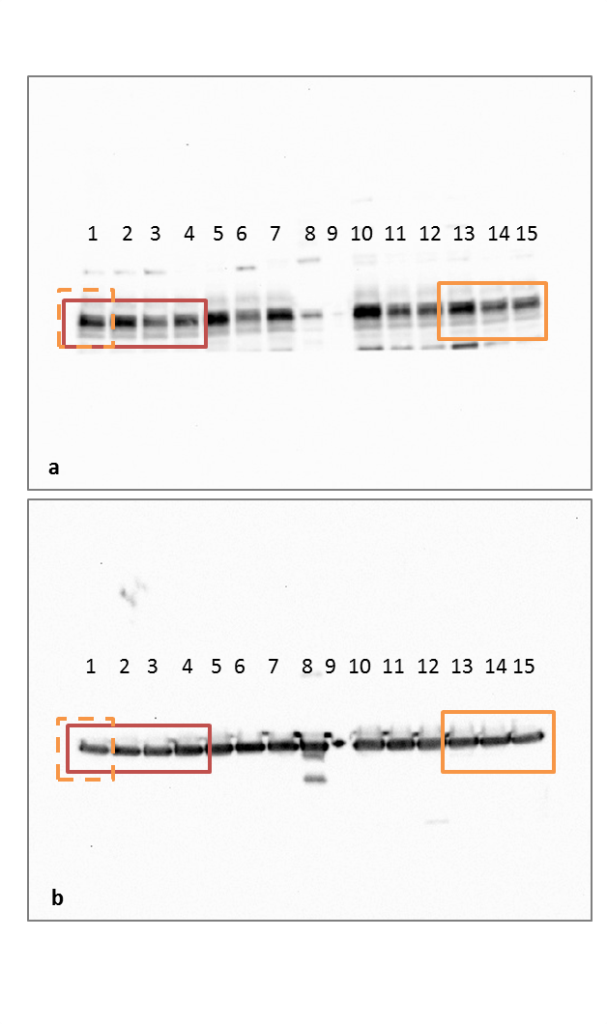


**Supplementary Information 6 Uncropped Western Blots used for Supplementary Figure 2e.** Blot against LpPLA_2_ **(a)** and corresponding β-Actin **(b).** Lanes from 1 to 15: 1. untreated control; 2. 200pg/ml IL-4; 3. 600pg/ml IL-4; 4. 1000pg/ml IL-4; 5. 200pg/ml IL-4; 6. 600pg/ml IL-4; 7. 1000pg/ml IL-4; 8. THP-1 (internal control); 9. Page Ruler (marker); 10. 200pg/ml IL-13; 11. 600pg/ml IL-13; 12. 1000pg/ml IL-13; 13. 200pg/ml IL-13; 14. 600pg/ml IL-13; 15. 1000pg/ml IL-13. The red frame indicates the cropped area for IL-4, the orange frame indicates the cropped area for IL-13, red-orange dashed frame indicates the shared untreated control on this membrane.
